# Fungal Cell Wall and Methyl-β–Cyclodextrin Synergistically Enhance Paclitaxel Biosynthesis and Secretion in *Corylus avellana* Cell Suspension Culture

**DOI:** 10.1038/s41598-020-62196-4

**Published:** 2020-03-25

**Authors:** Siamak Farhadi, Ahmad Moieni, Naser Safaie, Mohammad Sadegh Sabet, Mina Salehi

**Affiliations:** 1 Department of Plant Genetics and Breeding, Faculty of Agriculture, Tarbiat Modares University, Tehran, P.O. Box: 14115-336 Iran; 2 Department of Plant Pathology, Faculty of Agriculture, Tarbiat Modares University, Tehran, P.O. Box: 14115-336 Iran

**Keywords:** Plant biotechnology, Plant biotechnology, Secondary metabolism, Secondary metabolism

## Abstract

Paclitaxel is the top-selling chemotherapeutic drug used for the treatment of lung, ovarian and breast cancer as well as Kaposi’s sarcoma. Cell suspension culture (CSC) of *Corylus avellana* has been addressed as a promising alternative for producing paclitaxel. In this study, endophytic fungus strain YEF_33_ was isolated from *Taxus baccata* and identified as *Coniothyrium palmarum*. The effects of the elicitors derived from this fungus including cell extract, culture filtrate and cell wall (CW) and also chitin, alone or in combination with Methyl-β-Cyclodextrin (MBCD), on paclitaxel biosynthesis in *C. avellana* CSC were assayed for the first time. CW of *C. palmarum* was the most efficient fungal elicitor for paclitaxel biosynthesis in *C. avellana* CSC. The results revealed that MBCD affected paclitaxel biosynthesis differently depending on fungal elicitor type and vice versa. MBCD, either alone or in combination with fungal elicitors, induced a high secretion of paclitaxel, suggesting the decrement of toxicity and retro-inhibition processes of paclitaxel for cells. The joint effects of *C. palmarum* CW (2.5% (v/v) on 17^th^ day) and 50 mM MBCD synergistically enhanced paclitaxel biosynthesis (402.4 µg l^−1^; 5.8-fold), 78.6% of which (316.5 µg l^−1^) were secreted into culture medium, a level 146% higher than that in control.

## Introduction

Paclitaxel, the most effective chemotherapy agent against lung, ovarian and breast cancer, and also Kaposi’s sarcoma^1^, was originally extracted from *Taxus brevifolia* bark in 1967^2^ and its structure was published in 1971^3^, and then it was joined the drug development program of National Cancer Institute (NCI)^4^. Since the bark harvest is mortal for the trees, *T. brevifolia* was set on the endangered species list^4,5^. Plant cell suspension culture (CSC) is a hopeful and nature-friendly system to mass production of paclitaxel^6–8^. The worldwide demand for paclitaxel is rising at a high speed and its biosynthesis via *Taxus* cell factories is inadequate to handle the growing need of this medicine, mostly because of *Taxus* recalcitrant manner under *in vitro* conditions^6,7,9,10^. Thus, finding the alternative sources of this valuable secondary metabolite is prompted.

*Corylus avellana*, common hazel, has likewise been reported as a paclitaxel-producing species among angiosperms^6,7,10–15^. The major superiority of producing paclitaxel by *C. acellana* cell culture is that *in vitro* culture of *C. avellana* is more facile than that of *Taxus*^6,7,9,12,16,17^. *In vitro* culture of *C. avellana* has been reported as a hopeful and inexpensive method for producing paclitaxel^6,7,10,12,18^. High-yielding *in vitro* culture setup is essential for producing secondary metabolites through plant cell culture^19^. Bioactive compounds are usually fluctuated quantitatively/qualitatively under different conditions either *in vivo* or *in vitro*^6,7,10,12,20–23^. Even engineered plant cells for overexpressing key genes still need using the elicitors for mass- biosynthesis of relevant secondary metabolite. Thus, screening the efficient elicitors for stimulating the biosynthesis of secondary metabolite in a plant cell culture system is vital^24^. Amongst the various elicitors, fungal elicitors because of their high effectiveness and low cytotoxicity are mainly used for inducing the biosynthesis of secondary metabolites in plant cell cultures^25^.

The first defense line of plants is the recognition of specific conserved molecules of the microbes known as microbe-associated molecular patterns (MAMPs). The receptors localized on plant cell surface recognize MAMPs; this is the first defense induction phase which is known MAMP-triggered immunity^26–28^. Chitin is one of fungal MAMPs^29^ and induces the biosynthesis of different secondary metabolites in plant cell cultures^30–33^. Chitin forms a small percentage of fungal cell wall while function as a strong elicitor of plant defense system^34^. Previous research^35^ suggested that fungal cell wall stimulated the biosynthesis of phenylpropanoid derivatives in hairy root culture of *Linum album*. It is noteworthy that the informational fragments released from fungal cell wall through enzymatic degradation function as the signals for activating the genes involved in defensive chemical production^36^. Chitin, an important MAMP in plants, is hydrolyzed via plant chitinases and then short oligomers act as the signaling component for triggering plant defense response^34^.

Our previous studies^7,10^ showed that cell extract and culture filtrate of endophytic fungi enhanced paclitaxel biosynthesis in *C. avellana*. Nevertheless, no data is available respecting the effects of chitin, fungal cell wall and also comparing the efficiency of different fungal elicitors on paclitaxel content enhancement in *C. avellana* CSC. Fungal elicitor type, its concentration and adding-time, and also the exposure time of cell culture with it affected the paclitaxel biosynthesis in *C. avellana* CSC^7,10^. The optimal selection of these factors would set the scene for significant biosynthesis of paclitaxel by *C. avellana* cell culture.

The combined use of biotic and abiotic elicitors in *Taxus*^37^ and *Corylus avellana*^38^ CSCs has been shown to highly enhance the biosynthesis of paclitaxel.

Cyclodextrin has recently absorbed remarkable attention not only as an agent inducing the biosynthesis of secondary metabolites in plant cell cultures, the consequence of defense response induction, but also for its capability to constitute the inclusion complexes with poorly water-soluble apolar compounds and facilitate the secretion of metabolites from cell to culture medium, thus act as a genuine elicitor^39–41^. Some studies^40,42,43^ have been indicated that Methyl-β-Cyclodextrin (MBCD) enhanced paclitaxel biosynthesis, and also its secretion from cells to culture medium in *Taxus* cell culture. Therefore, the exploration of the combined effect of fungal elicitors with the elicitor/secretion activator MBCD on the biosynthesis and secretion of paclitaxel in *C. avellana* CSC is considered as crucial.

The main objective of this study was to enhance paclitaxel biosynthesis and also its secretion from cells to culture medium in a promising new biotechnological platform founded on *C. avellana* cell culture by optimizing elicitors. For achieving this purpose, potent new fungal elicitors such as cell wall and also MBCD were assayed for the first time in *C. avellana* CSC, either individually or as a combined treatment.

## Results and Discussion

### Identification of endophytic fungus

**Figure 1:**
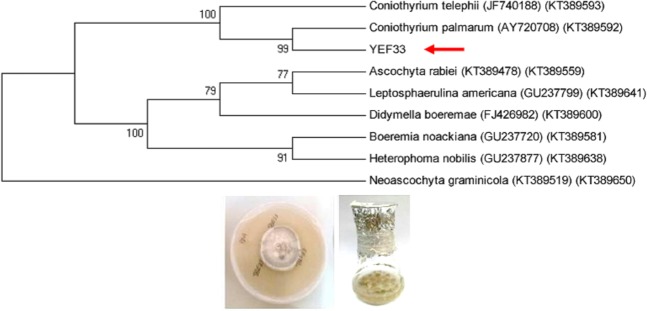
Molecular identification of strain YEF_33_ based on the analysis of the sequences of ITS1-5.8S-ITS2 region and *RPB2* gene.

Strain YEF_33_ was isolated from the inner bark of *T. baccata* and identified as *Coniothyrium palmarum* by analysis of the sequences of ITS1-5.8S-ITS2 region and *RPB2* gene (Fig. 1). *Coniothyrium* species contain very few helpful morphological features of taxonomic relationship^44^. This is the first report of this endophytic fungus on *T. baccata* tree. The partial sequences of ITS rDNA and *RPB2* obtained from *C. palmarum* strain YEF_33_ was deposited in GenBank (NCBI) under accession numbers MK530082 and MT113119, respectively.

### Effects of elicitors on *C. avellana* cell growth

**Figure 2:**
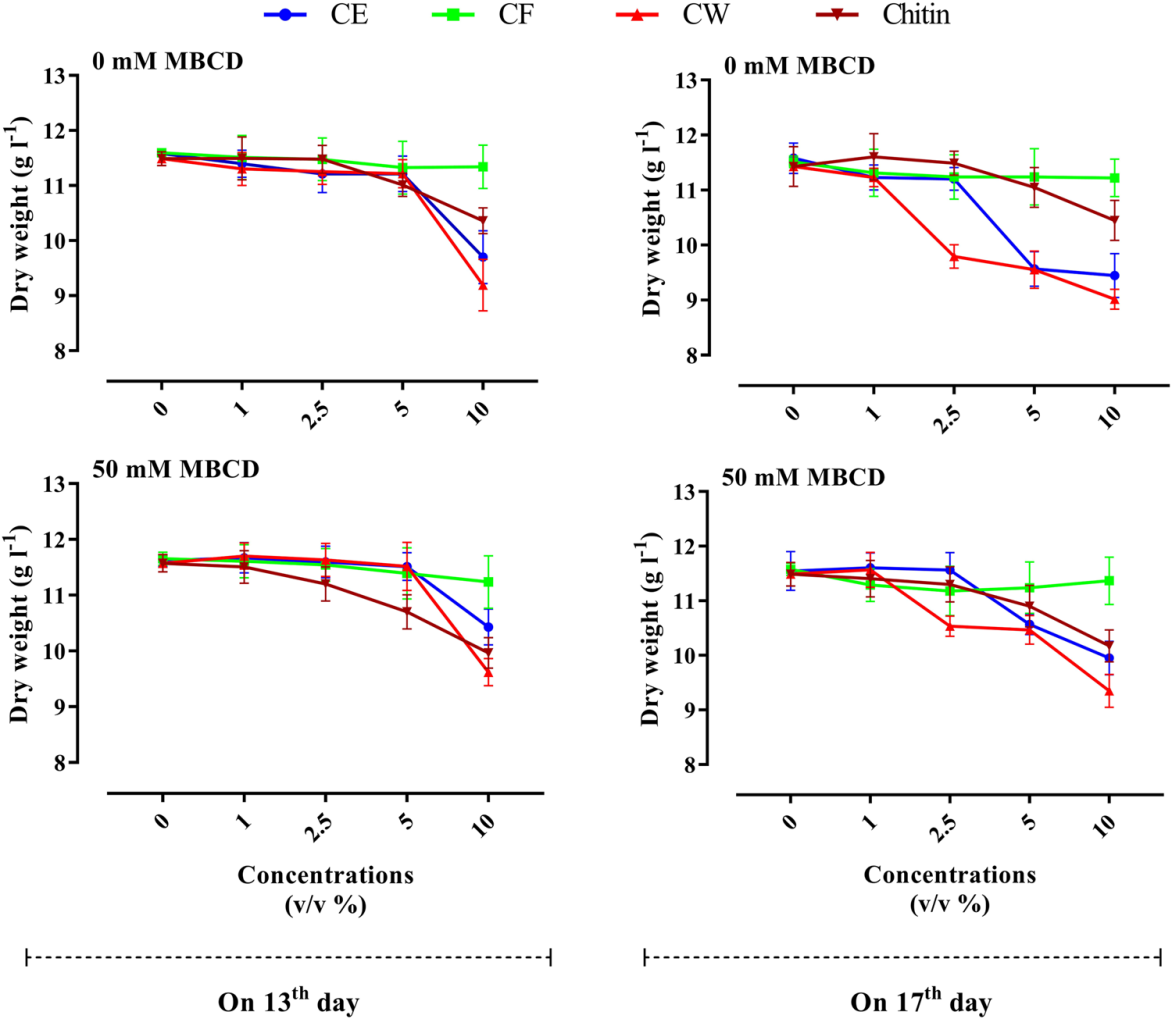
Effects of adding cell extract (CE), culture filtrate (CF) and cell wall (CW) of *Coniothyrium palmarum* and also chitin on 13^th^ (**a**) and 17^th^ (**b**) days of culture cycle, either individually or as a combined treatment with 50 mM of Methyl- β –Cyclodextrin (MBCD), on cell growth of *Corylus avellana* L. Average values are given, standard error are represented by vertical lines.

**Figure 3:**
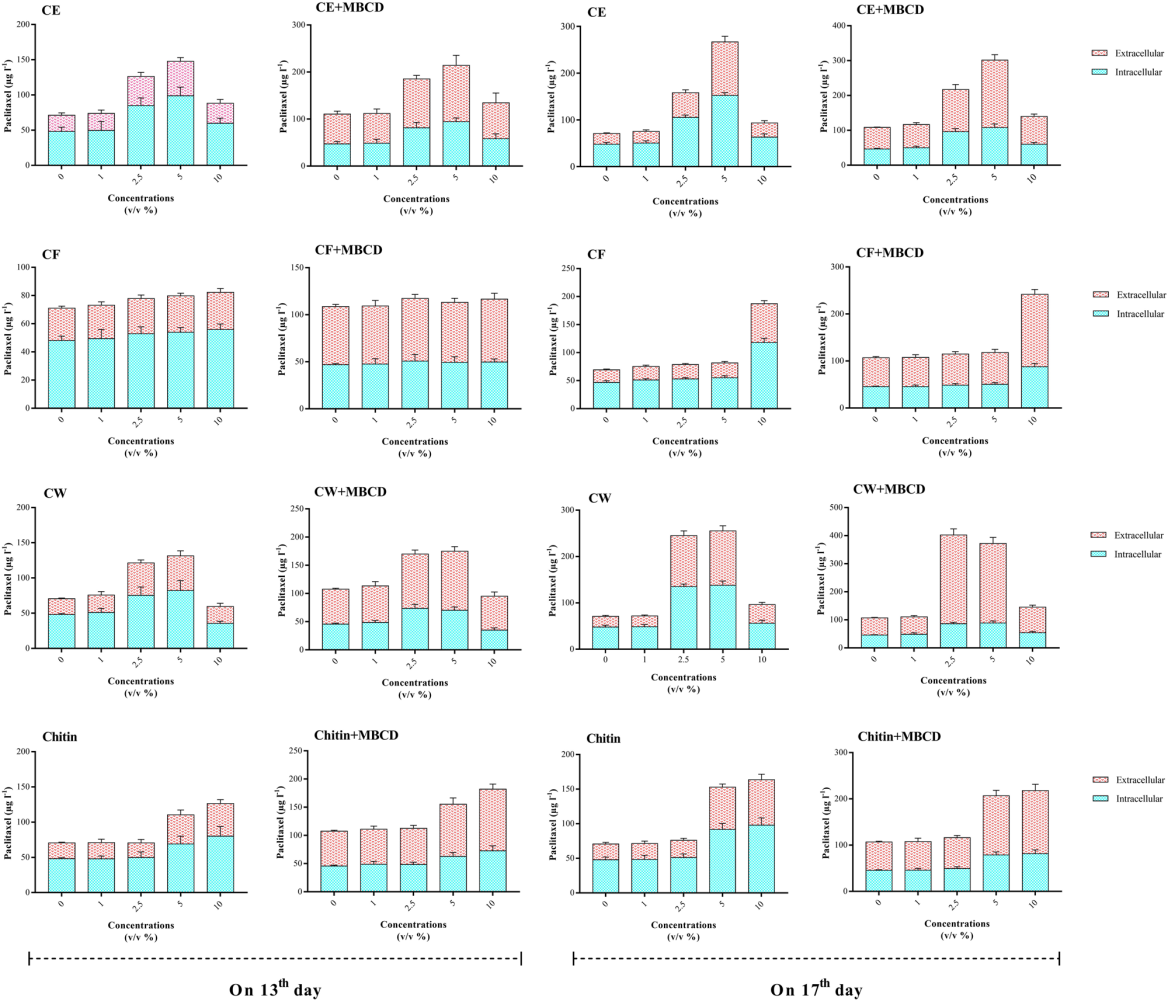
Effects of adding cell extract (CE), culture filtrate (CF) and cell wall (CW) of *Coniothyrium palmarum* and also chitin on 13^th^ and 17^th^ days of culture cycle, either individually or as a combined treatment with 50 mM of Methyl- β –Cyclodextrin (MBCD), on paclitaxel production in *Coryllus avellana* cell suspension culture. Average values are given, standard error are represented by vertical lines.

Analysis of variance (ANOVA) displayed that the main effects of the examined factors (MBCD, fungal elicitor type, concentration level and elicitor-adding day) and reciprocal interactions of MBCD × fungal elicitor type; fungal elicitor type × concentration level and also fungal elicitor type × elicitor-adding day on DW were significant (Table S1). The significant interaction effect of fungal elicitor type (CE, CF, CW and chitin) and concentration level showed that the effect of elicitors on cell growth was concentration level-dependent. Meanwhile, the significant interaction effect of fungal elicitor (CE, CF, CW and chitin) and elicitor-adding time (mid and late log phase) indicated that fungal elicitor type affected cell growth differently depending on elicitor-adding time. By reason of these significant interactions, the effects of fungal elicitor type were surveyed on each adding time and concentration level of elicitors. Means comparison showed that adding 1 and 2.5% (v/v) CE of *C. palmarum* on 13^th^ and 17^th^ days of cell culture cycle to *C. avellana* CSC did not significantly affect the cell growth, whereas adding 10% (v/v) of this elicitor in mid (day 13) and late (day 17) log phase significantly reduced DW as compared with control (Fig. 2). Cell culture treated with 10% (v/v) CE displayed an average growth rate of 0.456 g l^−1^ day^−1^, i.e. 16.4% lower than that of control (0.545 g L^−1^ day^−1^). Cell growth inhibition in cell culture exposed with 10% (v/v) CE seems to be as a result of CE toxicity at high concentration. As shown by Fig. 2, adding 5% (v/v) CE on 13^th^ day of culture cycle did not affect cell growth. However, adding 5% (v/v) of this elicitor at day 17 significantly repressed cell growth (Fig. 2). Average growth rate in CSC treated with 5% (v/v) CE of *C. palmarum* on 17^th^ of cell culture cycle was 0.456 g l^−1^ day^−1^, about 16.7% lower than that in control culture. It is noteworthy that *C. avellana* CSC exposed with 5% (v/v) CE on 17^th^ day exhibited higher paclitaxel biosynthesis than that on 13^th^ day (Fig. 3). The negative relation between paclitaxel accumulation and cell growth has been reported previously^12,45^. Also, the studies reported that high paclitaxel producing cell cultures can display cell growth inhibition^46,47^.

As shown by Fig. 2, adding the different concentrations of *C. palmarum* CF, and also 1, 2.5 and 5% (v/v) chitin to *C. avellana* CSC in mid and late log phase did not significantly affect cell growth, whiles adding 10% (v/v) chitin to CSC significantly reduced cell growth as compared with control. Cell culture exposed with 10% (v/v) chitin (0.495 g l^−1^ day^−1^) displayed a decrement of 8.9% in average growth rate as compared with control (0.544) (Fig. 2). Also, cell cultures exposed with 10% (v/v) CW on 13^th^ day, and also 2.5, 5 and 10% (v/v) CW of *C. palmarum* on day 17 displayed an average growth rate of 0.447 g l^−1^ day^−1^, i.e. 17.8% lower than that of control (0.544 g l^−1^ day^−1^) (Fig. 2). Cell growth inhibition in cell culture exposed with 10% (v/v) CW in mid and late log phase can be as a result of CW toxicity at high concentration. Given that *C. avellana* CSC exposed with 2.5 and 5% (v/v) CW on 17^th^ day exhibited significantly higher paclitaxel biosynthesis than that on 13^th^ day (Fig. 3), cell growth decrement in CSC subjected to mentioned treatment at day 17 can be attributed to reverse relation between paclitaxel accumulation and cell growth. Significant interaction effect of MBCD × fungal elicitor type (Table S1) showed that MBCD affected cell growth differently depending on fungal elicitor type and vice versa (i.e. fungal elicitors affected cell growth differently depending on presence or absence of MBCD) (Fig. 2). Average growth rate was not significantly influenced by MBCD alone, as has likewise been reported in *Vitis vinifera*^48^ and *Taxus* × *media*^42^. As mentioned above, adding 5% (v/v) CE, and also 2.5 and 5% (v/v) CW on 17^th^ day significantly repressed cell growth (Fig. 2). However, the presence of MBCD in culture medium significantly reduced the negative effect of adding 5% (v/v) CE, and also 2.5 and 5% (v/v) CW on 17^th^ day (Fig. 2), as it was also observed that pre-treatment of *Taxus* CSCs with MBCD decreased the negative effect of methyl jasmonate^42^ and coronatine^40^ on cell growth. Adding 5% (v/v) CE, and also 2.5 and 5% (v/v) CW on 17^th^ day to cell culture previously treated with MBCD resulted in cell growth increment of 9.2% as compared with that not treated with MBCD (Fig. 2). Indeed, MBCD constitute inclusion complexes with paclitaxel and other taxanes, thus boosting their secretion from cells to culture medium, also reducing cellular toxicity^42^. The effect of MBCD on decreasing the negative effect of mentioned treatment (adding 5% (v/v) CE, and also 2.5 and 5% (v/v) CW on 17^th^ day) can be attributed to secretion increment of taxanes to culture medium, and decreasing cellular toxicity.

### Effect of exposure period of fungal elicitors on paclitaxel content

To figure out the relevance between paclitaxel content and exposure period of fungal elicitors, the contents of paclitaxel in *C. avellana* CSCs treated with four concentrations (1, 2.5, 5 and 10% (v/v)) of CE, CF, CW and chitin in mid and late log phase were determined in 2-day periods after elicitation (Fig. S1). Generally, the increment of paclitaxel biosynthesis was observed throughout the period of cell growth and its maximum significant level was determined at day 21 (Fig. S1). Decreasing paclitaxel biosynthesis after 21^st^ day in non-treated cell culture with MBCD could be ascribed to enzymatic degradation of paclitaxel. However, *C. avellana* CSCs treated with MBCD exhibited no significant differences in paclitaxel produced on 21^st^ and 23^rd^ days (Fig. S1). It is noteworthy that MBCD forms the inclusion complexes with paclitaxel and inhibit its possible enzymatic degradation^42^. This could explain why paclitaxel content in CSCs treated with MBCD displayed no significant difference at days 21 and 23. Since maximum significant contents of paclitaxel were measured on day 21, this time was considered as the benchmark of paclitaxel biosynthesis in CSCs.

### Paclitaxel biosynthesis in elicited cell suspension cultures

The effects of CE, CF and CW of *C. palmarum*, as well as chitin on paclitaxel content were studied in a concentration level-, elicitor-adding time-dependent way, either individually or as a combined treatment with MBCD. The results of eliciting paclitaxel biosynthesis in *C. avellana* CSC using elicitors disclosed that intracellular, extracellular and total yield of paclitaxel were significantly affected by the mentioned elicitors (Table S1). The main effects of measured factors (MBCD, fungal elicitor type, concentration level and adding time of fungal elicitors) and their interactions (reciprocal and trilateral effects) except MBCD × concentration level, MBCD × fungal elicitor-adding time, MBCD × fungal elicitor type × concentration level, and MBCD × concentration level × fungal elicitor-adding time on total yield of paclitaxel were highly significant (p < 0.01) (Table S1). The significant interactions of fungal elicitor type and concentration level and adding time of fungal elicitors (Table S1) displayed that the concentration level and adding time of fungal elicitors impressed paclitaxel content differently at each fungal elicitor type. Also, the significant interaction of MBCD × fungal elicitor type revealed that the fungal elicitors affected the content of paclitaxel differently depending on the presence of MBCD and vice versa (i.e. MBCD affected paclitaxel biosynthesis differently depending on fungal elicitor type). To carefully analyze these significant interactions, the fungal elicitors were further examined on each concentration level and adding time of fungal elicitors as well as the presence or absence of MBCD.

### Effects of concentration level and adding time of fungal elicitors on the biosynthesis of paclitaxel

Means comparison revealed that cell cultures exposed with 2.5 and 5% (v/v) CE and CW of *C. palmarum* on 13^th^ day of culture cycle displayed a slight increase in paclitaxel biosynthesis (Fig. 3). As indicated in Fig. 3, adding 2.5 and 5% (v/v) CE and also CW at day 17 led to significantly higher paclitaxel contents (1.2-, 1.8-, 2.0 and 1.9-fold, respectively) than that on day 13. The most total yield of paclitaxel in cell cultures exposed to *C. palmarum* CE (266.9 μg L^−1^) was obtained by using 5% (v/v) of this elicitor on 17^th^ day of cell culture cycle, about 3.9- fold that detected in control culture (Fig. 3). The contents of extracellular and intracellular paclitaxel in CSC exposed to 5% (v/v) CE on 17^th^ day were 114.5 μg L^−1^ (5.2-fold) and 152.4 μg L^−1^ (3.2-fold), respectively (Fig. 3). It is noteworthy that cell cultures exposed with 5% (v/v) CE and also 2.5 and 5% (v/v) CW of *C. palmarum* displayed no significant difference in paclitaxel content (Fig. 3).

The results displayed that adding the different concentrations of *C. palmarum* CF on 13^th^ day of cell culture cycle and also 1, 2.5 and 5% (v/v) of it on 17^th^ day did not significantly affect paclitaxel biosynthesis (Fig. 3). However, cell cultures treated with 10% (v/v) CF of *C. palmarum* on 17^th^ day exhibited a pronounced increment in paclitaxel biosynthesis (2.8-fold) than control, measured 187.1 μg L^−1^ (Fig. 3).

As illustrated in Fig. 3, adding chitin to cell culture in mid and late log phase only at concentration levels of 5 and 10% (v/v) significantly enhanced paclitaxel biosynthesis. No significant difference was observed between paclitaxel biosynthesis in CSCs exposed to the concentration levels of 5 and 10% (v/v) chitin (Fig. 3) and the optimal concentration of it was 5% (v/v). Cell cultures subjected to 5 and 10% (v/v) chitin at day 17 had paclitaxel productivity of 7.54 μg l^−1^ day^−1^, about 33.7% higher than that at day 13 (5.64 μg l^−1^ day^−1^) (Fig. 3).

Out of CSCs exposed to four concentrations of 1, 2.5, 5 and 10% (v/v) of fungal elicitors in mid (day 13) and late (day 17) log phase of cell culture cycle, the highest yield of paclitaxel was measured in cell cultures treated with 5% (v/v) CE and also 2.5 and 5% (v/v) CW of *C. palmarum* added at day 17 (Fig. 3). Out of these treatments, 2.5% (v/v) CW is preferable as less volume of fungal elicitor was added to cell culture.

The results clearly showed that fungal elicitors had remarkable effects on improving paclitaxel biosynthesis in *C. avellana* cell culture. Several fungal elicitors applied in this study led to different responses regarding the enhancement of paclitaxel biosynthesis. CW of *C. palmarum* strain YEF_33_, isolated from the inner bark of *T. baccata*, has been demonstrated to be the most impressive fungal elicitor for inducing paclitaxel biosynthesis in *in vitro* cell culture of *C. avellana*. The varied responses of plant cells to fungal elicitors in enhancing the biosynthesis of secondary metabolite as observed in our research for paclitaxel biosynthesis can be associated with specific interactions of fungi and plant cells^7,49^. The receptors localized on plant cell surface recognize fungal elicitors and transfer the information for motivating cell defense system^50^. The specific structure of receptors leads to specially distinguish the specific elicitors^7,51^. Accordingly, all fungal elicitors are unable to induce a cell culture, and the selection of an efficient elicitor for the most biosynthesis of a favorite product in a special cell culture is essential.

Taken together, our results show that the influences of fungal elicitors on paclitaxel biosynthesis are affected by fungal elicitor concentration levels and its adding time to cell culture. So, optimizing these factors is required for the maximum biosynthesis of paclitaxel. The influences of the mentioned factors on paclitaxel biosynthesis in *C. avellana* cell culture were also reported in the previous studies using the elicitors derived from another endophytic fungi^7,10^.

### Effects of cyclodextrin and fungal elicitors on paclitaxel biosynthesis

As shown in Fig. 3, *C. avellana* CSCs treated with MBCD, alone or in combination with fungal elicitors, significantly enhanced paclitaxel biosynthesis. Significant interaction effect of MBCD × fungal elicitor type (Table S1) showed that MBCD affected paclitaxel biosynthesis differently depending on fungal elicitor type and vice versa (i.e. fungal elicitors affected paclitaxel biosynthesis differently depending on the presence or absence of MCBD). *C. avellana* CSCs treated with MBCD alone or in combination with fungal elicitors displayed a considerable variation in improving paclitaxel biosynthesis, ranging from 13% to 64% (Fig. 3). The most efficient treatment for increment of paclitaxel biosynthesis in *C. avellana* cell culture showed to be the combined one of MBCD and 2.5% (v/v) CW added at day 17 (Fig. 3), 5.8-fold higher than in control. By comparison, the individual use of MBCD and CW induced paclitaxel biosynthesis only 1.5- and 3.5-fold higher than control, respectively. These results show a synergistic effect of MBCD and CW on paclitaxel biosynthesis in *C. avellana* cell culture. The previous studies reported the synergistic effect of MBCD and methyl jasmonate^42^ or coronatine^40^ on paclitaxel biosynthesis in *Taxus* cell culture, but this is the first report on the synergistic effect of MBCD and fungal elicitor (CW) on paclitaxel biosynthesis. Intra- and extracellular paclitaxel of MBCD-pretreated CSC exposed with 2.5% (v/v) of *C. palmarum* CW on 17^th^ day of cell culture cycle were 86.0 μg L^−1^ (1.8-fold) and 316.5 μg l^−1^ (14-fold), respectively. It is noteworthy that paclitaxel content in cell culture treated with 2.5 and 5% (v/v) CW displayed no statistically significant difference.

**Figure 4:**
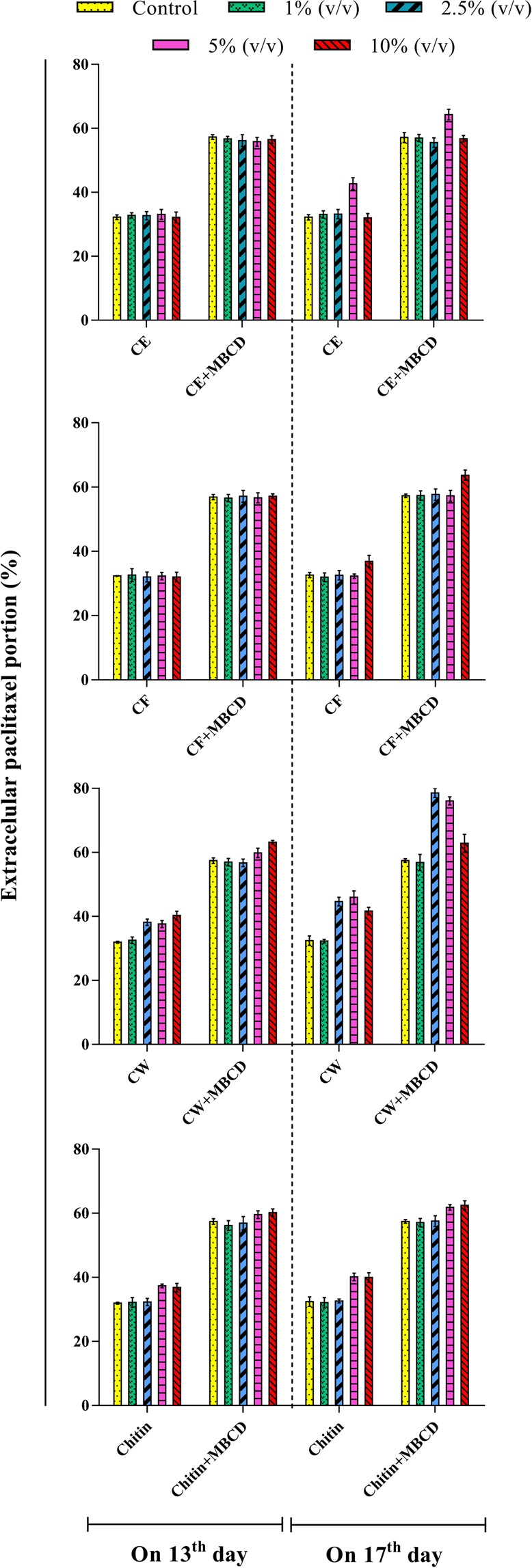
Extracellular paclitaxel portion in *Corylus avellana* cell suspension culture exposed with 1, 2.5, 5 and 10% (v/v) of cell extract (CE), culture filtrate (CF), cell wall (CW) and also chitin on 13^th^ and 17^th^ days of cell culture cycle, either individually or as a combined treatment with 50 mM of Methyl- β –Cyclodextrin (MBCD).

When examining cell capacity to secrete paclitaxel to the medium (Fig. 4), MBCD, either alone or in combination with fungal elicitors, induced a high secretion, whereas control and fungal elicitor-treated CSC maintained more than 50% of produced paclitaxel inside *C. avellana* cells. Facilitating paclitaxel secretion from cells into the medium in MBCD-pretreated CSCs was reported in previous studies^40,42^. MBCD, through the chemical structure, facilitates paclitaxel secretion from cells into the medium and mitigates feedback inhibition. Decreasing toxicity and retro-inhibition processes of paclitaxel could explain the high biosynthesis of paclitaxel found in CSC treated with MBCD and CW. Aforementioned synergistic effect on paclitaxel biosynthesis was not observed between MBCD and other fungal elicitors. It can be concluded that out of different fungal elicitors used in this study, only *C. palmarum* CW had high potential to induce paclitaxel biosynthesis, but the high accumulation of paclitaxel in cells led to feedback inhibition which is a drawback for its high biosynthesis. MBCD, due to paclitaxel secretion increment, declined the retro-inhibition processes and toxicity caused by paclitaxel accumulation in the cytoplasm and thus improved paclitaxel biosynthesis. Cell cultures treated with MBCD and fungal elicitors, individually and combined treatment, exhibited a remarkable variation in paclitaxel secretion, ranging from 31.8% to 78.6% (Fig. 4). Overall, MBCD-pretreated CSCs displayed a statistically significant increase in paclitaxel secretion as compared to control and also CSCs exposed with different fungal elicitors (Fig. 4). Out of different treatments, MBCD-pretreated cell culture exposed with 2.5 and also 5% (v/v) CW on 17^th^ day exhibited the best results (78.6 and 76.1, respectively) regarding extracellular paclitaxel portion, i.e.,137.4 and 70.7% higher than that in control and CSCs individually treated with 2.5 or 5% (v/v) CW. Cell capacity to secrete paclitaxel to the medium is essential for the commercial production because it enables continuous production with no destroying the cells and causes the extraction and purification processes to be easier and more economic.

The various treatments have been applied in *C. avellana* CSCs to enhance paclitaxel productivity. *C. avellana* CSC treated with the combined treatment of ultrasound (40 kHz for 3 min at days 10 and 12) and 50 mg l^−1^ salicylic acid displayed a 14-folds increment in paclitaxel biosynthesis, while a significant decrement in cell growth was observed by salicylic acid^38^. Also, *C. avellana* CSC subjected with ultrasound (29 KHz for 20 min) produced 6.07 mg kg^−1^ paclitaxel^52^. In another report, a slight increase in paclitaxel biosynthesis displayed in *C. avellana* cell culture affected by silver nano particles^53^. Gallego *et al*.^54^ also reported that coronatine highly induced paclitaxel biosynthesis, but strongly reduced cell growth in *C. avellana* CSC. In another attempt to find an efficient treatment, a combined treatment of salicylic acid and dibutyl phthalate highly enhanced paclitaxel biosynthesis in *C. avellana* CSC with displaying a synergistic effect, but these treatments decrease cell viability^55^. Also, the addition of benzoic acid to *C. avellana* CSC resulted in a 4-fold in paclitaxel biosynthesis^56^. In another research, Rahpeyma *et al*.^15^ displayed that the joint effects of phenylalanine (3 μM) and vanadyl sulfate (0.05 and 0.1 mM) in culture medium completed with fructose (3% (v/v)) led to a 2.3-fold increment in paclitaxel biosynthesis.

In the light of remarkable positive effect of ultrasound on paclitaxel biosynthesis without the negative effect on cell growth^38^, it can be suggested to evaluate the effects of ultrasound, CW and MBCD, either individually or in a combined treatment with each other using the factorial arrangement.

## Conclusion

Out of the elicitors evaluated in this study, the joint effect of *C. palmarum* CW (2.5% (v/v) on 17^th^ day) and 50 mM MBCD resulted in the highest stimulation of paclitaxel biosynthesis in *C. avellana* CSC. Although cell growth was decreased by about 8%, the total yield of paclitaxel was improved by 480% as compared with control. Indeed, *C. palmarum* CW is an efficient elicitor for paclitaxel biosynthesis in *C. avellana* CSC, although the presence of MBCD synergistically enhanced paclitaxel biosynthesis. Also, among the various elicitors, adding 2.5% (v/v) CW of *C. palmarum* on 17^th^ day to cell culture pre-treated with MBCD displayed the best results regarding extracellular paclitaxel portion (78.6%). The secretion of paclitaxel from cells into culture medium indubitably facilitates its extraction and the purification for paclitaxel production at the commercial level. Overall, the results show the potential of *C. avellana* CSC as a promising alternative for paclitaxel production, though this eco-friendly system yet needs the optimization.

## Material and Methods

### Isolation of endophytic fungi

Healthy samples of the stem, bud, bark pieces, and leaves were collected from *T. baccata* grown in Iran, in July, August, and September 2014. The surface sterilization of the samples was performed as described by Salehi *et al*.^7,12^. The surface sterilized pieces of stem, bud, bark, and leaves segments were excised and placed on PDAC (Potato Dextrose Agar (PDA); supplemented with 250 mg l^−1^ Chloramphenicol) in unique Petri dishes (100 × 15 mm), incubated at 25 °C to growth endophytic fungi. The isolates were purified by hyphal tip culture^57^. All fungal isolates were numbered as YEF# series and maintained on PDA at 4 °C.

### Molecular identification of endophytic fungus

Our group recently evaluated the effects of Cell extract (CE) and culture filtrate (CF) of a number of fungal endophytes isolated from *T. baccata* and *C. avellana* on the biosynthesis of paclitaxel in *C. avellana* cell culture^7,10^. CE of *Chaetomium globosum*^7^ and strain YEF_33_ were selected as the most impressive elicitors for stimulating paclitaxel biosynthesis in *C. avellana* CSC. *C. avellana* CSCs exposed with 10% (v/v) *C. globosum* CE and 5% (v/v) CE of strain YEF_33_ displayed no significant difference in paclitaxel production. Given that the elicitation effect of CE of strain YEF_33_ was stronger than that of *C. globosum*, the strain YEF_33_ was used in this study.

Strain YEF_33_ was cultured in potato dextrose broth (PDB) and maintained in a shaker incubator at 110 rpm and 25 °C for 7 days. The mycelia were harvested; freeze-dried and then genomic DNA extraction was performed as described by Salehi *et al*.^7,12^. ITS fragments were amplified using universal primers ITS1 and ITS4 (White *et al*.)^58^, *RPB2 using* fRPB2-5F and fRPB2-7cR primers (Liu *et al*.)^59^ (Table S2). PCR reaction mixtures (25 µl) consisted of 1 µl genomic DNA (~100 ng), 1 µl forward and reverse primers (10 pM), and 12.5 µl Premix Taq (TaKaRa Biotechnology Ltd., Japan), and 10.5 µl PCR quality water. PCR reaction programs were an initial denaturation at 94 °C for 3 min, followed by 30 cycles of denaturation (94 °C for 30 s), annealing (56 °C (ITS) and 55 °C (RPB2) for 30 s), extension (72 °C for 1 min) and a final extension at 72 °C for 5 min. PCR products analysis and purification, sequencing and phylogenetic analysis were made as described previously^7,12^.

### Cell suspension culture

Callus of *C. avellana* (ecotype Gerd Ashkorat) was produced from seed cotyledons on MS medium^60^ supplemented with 2 mg l^−1^ 2, 4-D and 0.2 mg l^−1^ BAP, and 8 g l^−1^ agar agar^6^. CSC of *C. avellana* was obtained as described by Salehi *et al*.^6,7,10,12^.

### Elicitor preparation

CE and CF elicitors were prepared as described previously^7^. The isolation of cell wall of strain YEF_33_ was performed as described by Prados‐Rosales *et al*.^61^, with minor modifications. Seven-day-old mycelia of strain YEF_33_ grown in potato dextrose broth (PDB) medium on a gyratory shaker at 110 rpm in darkness at 25 °C were harvested by filtration and rinsed three times with double distilled water. Then the freeze-dried mycelia were crushed in liquid nitrogen, and soaked in a buffer containing 10 mM Tris–HCl with pH 7.5, 5 mM dithiothreitol, and 1 mM phenylmethanesulfonyl fluoride (PMSF), and mixed thoroughly. The suspension was partitioned into a cell wall portion (pellet) and a soluble cytoplasmic portion (supernatant) by centrifuging at 10,000 g for 15 min. Then, fungal cell wall pellet was washed five times with deionized water supplemented with 1 M NaCl and 1 mM PMSF, and then washed five times with ice-cold water supplemented with 1 mM PMSF. Finally, the crushed cell wall was soaked in deionized water including 1% (v/v) acetic acid (1 mg ml^−1^), mixed well, and incubated at 50 °C for 2 h. Then the mixture was filtered through 0.22 μm cellulose acetate syringe filters and designated as cell wall (CW).

### Elicitation experiment

For elicitation, 1.5 ± 0.1 g of *C. avellana* cells (fresh mass) was cultured in 100 ml flasks having 30 mL MS medium supplemented with 2 mg l^−1^ 2,4-D and 0.2 mg l^−1^ BAP and then elicited with 50 mM MBCD, either individually or a combined treatment with fungal elicitors (CE, CF, CW and chitin). It is noteworthy that MBCD was added to culture medium before autoclaving.

Based on the preliminary experiment, four concentrations (1, 2.5, 5 and 10% (v/v)) of fungal elicitors “CE, CF, CW and chitin”, and also mid (day 13) and late (day 17) log phase were elected for adding fungal elicitors. Control received an equal volume of water (for CE)/ PDB (for CF)/ water including 1% (v/v) acetic acid (for CW and chitin). The growth curve of *C. avellana* cells has been given elsewhere^6^.

### Cell growth measurement

Cell growth was defined by the measurement of cell dry weight (DW). Cell biomass was separated from culture medium by the filtration (Whatman No. 1) and rinsed with distilled water for eliminating the residual medium, afterward freeze-dried to constant weight by a vacuum-freeze drier.

### Quantification of paclitaxel

*C. avellana* cells were separated from culture medium by a filter paper (Whatman No. 1). Extracellular and intracellular paclitaxel were extracted from the cells and culture medium using a procedure described by Salehi *et al*.^6,7,12^. Filtering all samples was performed by 0.22 µm cellulose acetate syringe filters before HPLC analysis. Paclitaxel in the samples were analyzed by HPLC (Waters, USA) with a C18 analysis column (MachereyeNagel EC 250/4.6 Nucleodur). Each sample (20 µl) was injected and detected at 230 nm using a UV detector. The mobile phase was methanol: water (80:20 v/v) at a flow rate of 1.0 ml/min. The quantification of paclitaxel was based on an external standard of genuine paclitaxel (Sigma) (Figs. S2 and S3).

### Statistical analysis

The experiment was conducted as factorial based on a complete randomized block design (CRBD). The factorial arrangement of the treatments consisted of four factors containing MBCD with two levels (0 and 50 mM), elicitor type with 7 levels (CE, CF, CW, chitin, water, PDB and water including 1% (v/v) acetic acid), the concentration level of elicitor with four levels (1, 2.5, 5 and 10% (v/v)) and elicitor-adding time with two levels (mid and late log phase), given 112 treatments.

The experiment was conducted in triplicate. The normality and equal variance hypotheses were met and conventional parametric statistics were applied for the analysis. The data was analyzed using analysis of variance (ANOVA) and mean comparisons were performed by least significant difference (LSD) using SAS (SAS 9.1) and SPSS (SPSS 15.0). Term “significant” indicates the differences for P < 0.05. GraphPad Prism (GraphPad Prism 5) software was used for making graphs.

### Declarations

All authors approve Ethics and consent for participation and publication.

All authors of the manuscript have read and agreed to its content and are accountable for all aspects of the accuracy and integrity of the manuscript in accordance with ICMJE criteria

That the article is original, has not already been published in a journal, and is not currently under consideration by another journal.

## Supplementary information

Supplementary Information.

## Data Availability

The dataset supporting the conclusions of this article is included in the article.
