# Supplementary material for: Fungal Cell Wall and Methyl-β–Cyclodextrin Synergistically Enhance Paclitaxel Biosynthesis and Secretion in Corylus avellana Cell Suspension Culture
Source: Sci Rep. 2020 Mar 25;10:5427. doi: 10.1038/s41598-020-62196-4 (PMC7096423; doi:10.1038/s41598-020-62196-4)
Supplement: Supplementary file 1 — Supplementary Information. [file 41598_2020_62196_MOESM1_ESM.docx]

## **Supplementary Information**

**Fungal Cell Wall and Methyl-β–Cyclodextrin Synergistically Enhance Paclitaxel biosynthesis and Secretion in *Corylus avellana* Cell Suspension Culture**

Siamak Farhadi^1^, Ahmad Moieni^1*^, Naser Safaie^2^, Mohammad Sadegh Sabet^1^, Mina Salehi^1^

^1^ Department of Plant Genetics and Breeding, Faculty of Agriculture, Tarbiat Modares University, Tehran, P.O. Box: 14115-336, Iran

^2^ Department of Plant Pathology, Faculty of Agriculture, Tarbiat Modares University, Tehran, P.O. Box: 14115-336, Iran

*Corresponding author: [moieni_a@modares.ac.ir](mailto:moieni_a@modares.ac.ir)

| **Table S1**  Analysis of variance for the effects of cyclodextrin, fungal elicitor type, their concentration levels and also adding fungal elicitors at days 13 and 17 of culture cycle on cell growth and paclitaxel biosynthesis of *Corylus avellana* cell suspension culture. | | | | | | | |  |
| --- | --- | --- | --- | --- | --- | --- | --- | --- |
|  |  | Mean squares | | | | | |  |
| Source of variation | Degree of freedom | Dry weight | Paclitaxel | | | | Extracellular paclitaxel portion | |
|  |  |  | Intracellular (μg g^-1^ DW) | Intracellular  (μg l^-1^) | Extracellular | Total |  |  |
| Block | 2 | 0.98^ns^ | 1.4^ns^ | 198.3^ns^ | 534.7^*^ | 1382.7^*^ | 18.6^*^ | |
| Cyclodextrin (A) | 1 | 1.41^*^ | 35.6^**^ | 2918.5^**^ | 213064.2^**^ | 165700.7^**^ | 50488.0^**^ | |
| Fungal elicitor type (B) | 6 | 7.70^**^ | 89.3^**^ | 7855.0^**^ | 19139.1^**^ | 48500.3^**^ | 289.3^**^ | |
| Concentration level (C) | 3 | 10.43^**^ | 60.5^**^ | 5723.4^**^ | 13251.6^**^ | 36428.8^**^ | 130.7^**^ | |
| elicitor-adding time (D) | 1 | 3.70^**^ | 70.2^**^ | 5270.8^**^ | 25878.5^**^ | 54273.3^**^ | 277.6^**^ | |
| A × B | 6 | 0.82^*^ | 4.9^**^ | 289.6^**^ | 2735.0^**^ | 1329.1^**^ | 6.9^ns^ | |
| A × C | 3 | 0.02^ns^ | 3.6^**^ | 261.2^*^ | 1574.0^**^ | 695.0^ns^ | 0.8^ns^ | |
| A × D | 1 | 0.03^ns^ | 10.9^**^ | 881.8^**^ | 3425.9^**^ | 860.7^ns^ | 12.6^ns^ | |
| B × C | 18 | 1.71^**^ | 28.4^**^ | 3225.8^**^ | 7429.1^**^ | 19877.6^**^ | 55.5^**^ | |
| B × D | 6 | 0.98^**^ | 14.2^**^ | 999.0^**^ | 7908.5^**^ | 14102.6^**^ | 65.2^**^ | |
| C × D | 3 | 0.38^ns^ | 9.0^**^ | 742.3^**^ | 3638.3^**^ | 7125.0^**^ | 41.0^**^ | |
| A × B × C | 18 | 0.03^ns^ | 2.1^**^ | 177.9^**^ | 1054.8^**^ | 446.2^ns^ | 3.7^ns^ | |
| A × B × D | 6 | 0.03^ns^ | 2.7^**^ | 185.4^*^ | 1999.9^**^ | 1102.0^**^ | 13.2* | |
| A × C × D | 3 | 0.06^ns^ | 2.0^*^ | 121.6^ns^ | 506.2^**^ | 191.7^ns^ | 4.6^ns^ | |
| B × C × D | 18 | 0.35^ns^ | 5.8^**^ | 520.6^**^ | 3083.1^**^ | 5761.8^**^ | 36.9^**^ | |
| A × B × C × D | 18 | 0.04^ns^ | 1.7^**^ | 121.7^ns^ | 699.6^**^ | 348.0^ns^ | 7.4^ns^ | |
| Error | 222 | 0.33 | 0.8 | 80.3 | 120.4 | 328.0 | 5.8 | |
| *, ** and ns indicate significant difference p<0.05, p<0.01 and non-significant, respectively. | | | | | | | |  |

| **Table S2**  Sequences of the primers used for the PCR analysis. | | | |
| --- | --- | --- | --- |
| Gene | Primers | Sequence (5′-3′ ) | Reference |
| ITS1-5.8S-ITS2 rDNA | ITS1 | TCCGTAGGTGAACCTGCGG | White et al., 1990 |
|  | ITS4 | TCCTCCGCTTATTGATATGC |  |
| RPB2 | fRPB2-5F | GAYGAYMGWGATCAYTTYGG | Liu et al., 1999 |
|  | fRPB2-7cR | CCCATRGCTTGYTTRCCCAT |  |

**Fig. S1.** Time course of paclitaxel biosynthesis in *Corylus avellana* cell cultures exposed with 1, 2.5, 5 and 10% (v/v) of cell extract (CE), culture filtrate (CF), cell wall (CW) and also chitin on 13^th^ and 17^th^ days of cell culture cycle, either individually or as a combined treatment with 50 mM of Methyl- β –Cyclodextrin (MBCD).

**
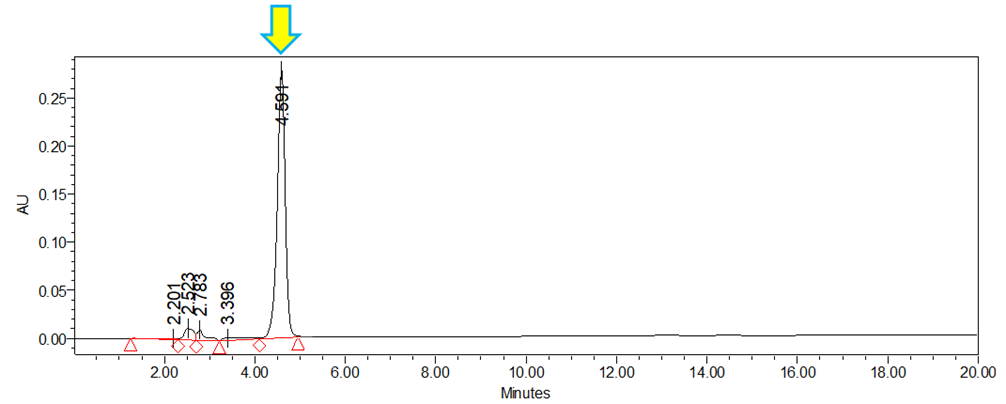
**

**Fig. S2.** HPLC chromatogram for paclitaxel standard (retention time: 4.59± 0.05 min).


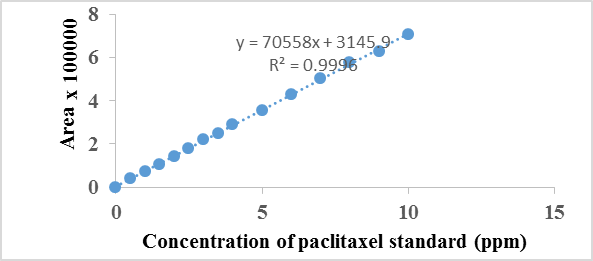


**Fig. S3.** Standard curve of paclitaxel.
